# Novel endosomal NOX2 oxidase inhibitor ameliorates pandemic influenza A virus‐induced lung inflammation in mice

**DOI:** 10.1111/resp.13524

**Published:** 2019-03-18

**Authors:** Eunice E. To, Raymond Luong, Jiayin Diao, John J. O’ Leary, Doug A. Brooks, Ross Vlahos, Stavros Selemidis

**Affiliations:** ^1^ School of Health Sciences and Biomedical Sciences RMIT University Melbourne VIC Australia; ^2^ Monash Institute of Pharmaceutical Sciences Monash University Melbourne VIC Australia; ^3^ Sir Patrick Dun's Laboratory, Central Pathology Laboratory St James's Hospital Dublin Ireland; ^4^ Emer Casey Research Laboratory, Molecular Pathology Laboratory The Coombe Women and Infants University Hospital Dublin Ireland; ^5^ CERVIVA Research Consortium Trinity College Dublin Ireland; ^6^ School of Pharmacy and Medical Sciences, Division of Health Sciences University of South Australia Adelaide SA Australia; ^7^ Department of Pharmacology, Infection and Immunity Program, Biomedicine Discovery Institute Monash University Melbourne VIC Australia

**Keywords:** endosome, inflammation, influenza, NOX2 oxidase, respiratory infections

## Abstract

**Background and objective:**

Influenza A viruses (IAV) cause respiratory tract infections that can be fatal when the virus spreads to the alveolar space (i.e. alveolitis), and this is mainly observed with highly pathogenic strains. Reactive oxygen species (ROS) production by the NOX2 NADPH oxidase in endosomes has been directly implicated in IAV pathology. Recently, we demonstrated that treatment with a novel endosome‐targeted NOX2 oxidase inhibitor, cholestanol‐conjugated gp91dsTAT (Cgp91ds‐TAT), attenuated airway inflammation and viral replication to infection with a low pathogenic influenza A viral strain. Here, we determined whether suppression of endosome NOX2 oxidase prevents the lung inflammation following infection with a highly pathogenic IAV strain.

**Methods:**

C57Bl/6 mice were intranasally treated with either DMSO vehicle (2%) or Cgp91ds‐TAT (0.2 mg/kg/day) 1 day prior to infection with the high pathogenicity PR8 IAV strain (500 PFU/mouse). At Day 3 post‐infection, mice were culled for the evaluation of airway and lung inflammation, viral titres and ROS generation.

**Results:**

PR8 infection resulted in a marked degree of airway inflammation, epithelial denudation, alveolitis and inflammatory cell ROS production. Cgp91ds‐TAT treatment significantly attenuated airway inflammation, including neutrophil influx, the degree of alveolitis and inflammatory cell ROS generation. Importantly, the anti‐inflammatory phenotype affected by Cgp91ds‐TAT significantly enhanced the clearance of lung viral mRNA following PR8 infection.

**Conclusion:**

Endosomal NOX2 oxidase promotes pathogenic lung inflammation to IAV infection. The localized delivery of endosomal NOX2 oxidase inhibitors is a novel therapeutic strategy against IAV, which has the potential to limit the pathogenesis caused during epidemics and pandemics.

## Graphical abstract

Lung inflammation to influenza A virus (IAV) infection can be fatal when the virus spreads to the alveolar space. We demonstrated that pathogenic inflammation to IAV is promoted by endosomally located NOX2 oxidase and that targeted inhibition of the reactive oxygen species (ROS) generated by this enzyme suppressed this inflammation and improved viral clearance.

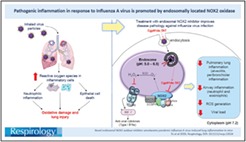

**See related** https://onlinelibrary.wiley.com/doi/10.1111/resp.13629

## INTRODUCTION

Influenza epidemics and pandemics result in significant global morbidity and mortality, and represent a major burden to healthcare systems. The current approaches to limit the pathogenesis associated with influenza virus infections are vaccines and antiviral drugs. Despite vaccines being relatively efficacious in containing the spread of influenza viruses in some years, the demand for mass production of vaccines in a timely manner and inaccuracies in predicting the antigenicity of seasonal circulating strains does reduce the effectiveness of the vaccination approach. Neuraminidase inhibitors have been used to interfere with newly formed virions to prevent the spread of the virus to neighbouring cells.^1^ However, resistance to this and other classes of drugs have also been demonstrated with some circulating strains of influenza virus.^2^, ^3^ This highlights the unmet need for the development of novel therapeutics that can be used against viruses irrespective of the strain and pathogenicity, and therefore be used to combat newly emerging strains of virus. This can potentially be achieved by targeting the over‐exuberant inflammatory response that the virus evokes in the host, and which it uses to affect optimal replication/spread and causes the significant pathogenesis.

Oxidative stress due to dysregulated reactive oxygen species (ROS) production and altered metabolism has been directly implicated in the pathogenesis of influenza virus infections.^4^, ^5^ The consensus is that excessive ROS response triggers redox‐sensitive pathways that contribute to the respiratory burst, causing inflammation and tissue injury. Therefore, targeting the primary source of ROS production in inflammatory cells, that is the NOX2‐containing NADPH oxidase to attenuate ROS generation, may be an effective means for reducing the ROS‐induced viral burden and tissue damage. To support this notion, the specific deletion of the NOX2 gene was associated with improved lung function and a reduced amount of lung tissue damage.^6^, ^7^, ^8^ Our recent findings revealed that the primary subcellular site of ROS generation to influenza virus infection is the endosome.^9^ We previously showed that influenza A virus (IAV) triggers ROS production within the endosome by: (i) internalizing into early endosome antigen‐1 (EEA1)‐positive endosomes by a clathrin‐coated pit‐dependent endocytic process; (ii) activation of endosomally located toll‐like receptor 7 (TLR7) by its single‐stranded RNA content; (iii) activation of protein kinase C that is downstream of TLR7 activation resulting in phosphorylation of the NOX2 oxidase, organizer protein p47phox; and finally (iv) the assembly of the NOX2 oxidase complex at the endosomal membrane releasing superoxide within the endosome lumen.^9^ Strikingly, we demonstrated that several seasonal and pandemic IAV strains each evoke elevated ROS production in endosomes and we therefore hypothesized that targeting endosomal compartments to limit ROS production could reduce the viral burden associated with influenza infections. We developed an innovative molecular targeting system to deliver a specific NOX2 oxidase inhibitor (cholestanol‐conjugated gp91ds‐TAT; Cgp91ds‐TAT) to dampen the ROS response and limit viral pathogenesis.^9^, ^10^ This innovative endosome‐specific targeting system provided proof‐of‐principle for limiting ROS production to restrict viral pathogenesis, using a mild influenza viral strain, that is Hong Kong H3N2 X‐31 strain.^9^ To advance the concept that Cgp91ds‐TAT blocks the pathology to IAV infection irrespective of pathogenicity, the present study aimed to examine the effect of endosomal NOX2 inhibition against the highly virulent PR8 strain of IAV. In striking contrast to the mild Hkx‐31 strain, PR8 causes a more profound lung inflammation that is characterized by a high degree of peribronchial inflammation and parenchymal inflammation or alveolitis. Here, we show that Cgp91ds‐TAT treatment 1 day prior to PR8 infection significantly reduced airway and lung inflammation, and in particular alveolitis, neutrophil infiltration and oxidative stress. This confirmed that endosomal ROS promotes a deleterious inflammatory immune response in the lung, following influenza virus infection, and that this may be exploited as a novel therapeutic target via local delivery of endosomal NOX2 oxidase inhibitors.

## METHODS

### Experimental animals

C57Bl/6J mice were obtained from Monash animal services (Monash University, Melbourne, Australia). Animals were housed in a high barrier facility designed for infectious experimental work with ad libitum access to water and standard rodent chow (4.8% fat and 0.02% cholesterol). All mice used in this study were males aged 8–12 weeks. This study was approved by the Animal Experimentation Ethics Committee of Monash University (ethics number: MARP/2016/024), and conducted in compliance with the guidelines of the National Health and Medical Research Council (NHMRC) of Australia or Australian Research Council (ARC) on animal experimentation.

### Virus infection models and treatment regimens

Mice were anaesthetized by isoflurane inhalation (2–5% isoflurane/95% oxygen air mixture) and then treated daily via intranasal administration with control DMSO (2%) or Cgp91ds‐TAT (0.2 mg/kg) 1 day prior to infections over a 4‐day period. We have previously shown that Cgp91ds‐TAT at 0.2 mg/kg/day abolished ROS production in the bronchoalveloar lavage fluid (BALF) inflammatory cells following infection with the H3N2 (Hk‐x‐31 strain of influenza^9^). Mice were intranasally infected with PBS control or PR8 IAV (500 PFU/mouse) and killed for assessment 3 days post‐infection. This dose of PR8 causes severe lung inflammation including widespread alveolitis, peribronchial inflammation and perivascular inflammation. At the end of each experiment, BALF was taken by a lavage technique and the left lung lobe was placed in 10% neutral buffered formalin for histological examination.^9^

### Airway inflammation and differential cell counts

Mice were killed by intraperitoneal (i.p.) injection of a mixture of ketamine/xylazene (100 mg/kg) and airway inflammation and differential cell counts were performed as previously described.^7^

### Histological analysis

The left lung lobe was incubated in 10% neutral buffered formalin at RT for 24–28 h prior to processing. After fixation, lung samples were embedded in paraffin wax and sections (3–4 μm thick) were cut (CM1850, Leica Microsystems, Wetzlar, Germany) longitudinally through the left lung lobe and placed on super frost slides (Menzel Gläser, Braunschweig, Germany). Sections were de‐waxed with xylene and a series of graded ethanol that were then stained with haematoxylin and eosin (HE; Sigma, St. Louis, Missouri, United States) and imaged by light microscopy. Samples were analysed on an Imagescope (Leica Biosystems, USA) and blindly scored from 0 to 5 for (higher numbers indicate increased disease severity). The lung injury score was determined by a grading system that combined assessments of alveolitis, inflammatory cell infiltration and peribronchiolar inflammation, as determined by two independent assessors.^11^ A score of 0 was indicative of healthy lungs (no damage); 1 indicated very mild damage; 2 mild damage; 3 moderate damage; 4 severe damage; and 5 represented extremely severe histological changes.

### L‐O12 enhanced chemiluminescence

NOX2 oxidase‐dependent ROS generation was measured using L‐012 enhanced chemiluminescence as previously described.^9^

### Viral mRNA expression via quantitative polymerase chain reaction

Viral RNA was measured as we have previously described.^9^

### Statistical and image analysis

Data for ROS generation, viral mRNA expression, total and differential cell counts were analysed using one‐way analysis of variance (ANOVA) followed by Tukey's post hoc multiple comparison test. All data were expressed as mean ± SEM. Histological lung samples were analysed on Imagescope (Leica Biosystems, Wetzlar, Germany) and statistical comparisons were made using the non‐parametric Kruskal–Wallis test, followed by the Dunn's post hoc test. All statistical tests were performed using GraphPad Prism (GraphPad Software Version 6.0, San Diego, CA, USA). A *P*‐value of <0.05 indicated significance.

### Chemicals

FBS (Sigma) was stored at −20°C in 50 mL aliquots. L‐012 (WAKO Chemicals, Osaka, Japan) and phorbol dibutyrate (PDB) (Sigma) were dissolved in DMSO (100%) and prepared as 10 mM stock solutions in aliquots of 10, 25 and 50 μL, and stored at −20°C. Custom‐designed peptide, Cgp91ds‐TAT, was dissolved in 100% DMSO in 5 mM (10 μL) aliquots and stored at −20°C.

## RESULTS

### Airway inflammation and cell differentials

**Figure 1:**
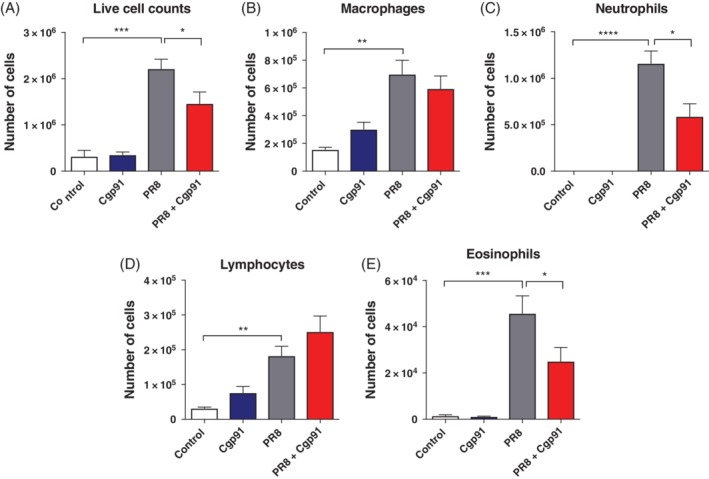
Cgp91ds‐TAT treatment suppressed BALF inflammation in mice infected with PR8 virus. Mice were treated daily with intranasal administration of Cgp91ds‐TAT (0.2 mg/kg) or DMSO (2%; control) over a 4‐day period. Mice were intranasally infected with PR8 (500 PFU) or PBS control 1 day post initial drug treatment. BALF inflammation was assessed via counting the total number of (A) live cells and differential cell counts of (B) macrophages, (C) neutrophils, (D) lymphocytes and (E) eosinophils. A total of 500 cells were counted from random fields by standard morphological criteria. Data are expressed as mean ± SEM (control, *n* = 7; Cgp91ds‐TAT, *n* = 5; PR8, *n* = 9; PR8 + Cgp91ds‐TAT, *n* = 10). Statistical analysis was conducted using one‐way ANOVA followed by Tukey's post hoc test for multiple comparison tests. Statistical significance was taken where *P* < 0.05. *P < 0.05; **P < 0.01; ***P < 0.001; ****P < 0.0001. ANOVA, analysis of variance; BALF, bronchoalveloar lavage fluid; Cgp91ds‐TAT, cholestanol‐conjugated gp91ds‐TAT; DMSO, dimethyl sulphoxide; PFU, plaque forming units.

To assess airway inflammation, the total number of live cells in the BALF was measured. There was a substantial increase in cellular infiltration in the PR8‐infected mice compared to the controls (Fig. 1A). This inflammatory response to PR8 infection was significantly less in mice treated with Cgp91ds‐TAT (Fig. 1A). Using differential cell counting, PR8 infection showed a significant increase in the number of BALF macrophages, neutrophils, lymphocytes and eosinophils when compared to the uninfected controls (Fig. 1B–E). Cgp91ds‐TAT treatment attenuated the numbers of neutrophils and eosinophils by approximately 40–50% (Fig. 1C, E); however, it did not alter macrophage or lymphocyte numbers following PR8 infection. Importantly, Cgp91ds‐TAT treatment of naïve‐uninfected mice had no effect on the cell populations examined (Fig. 1).

### Cgp91ds‐TAT attenuates pulmonary inflammation

**Figure 2:**
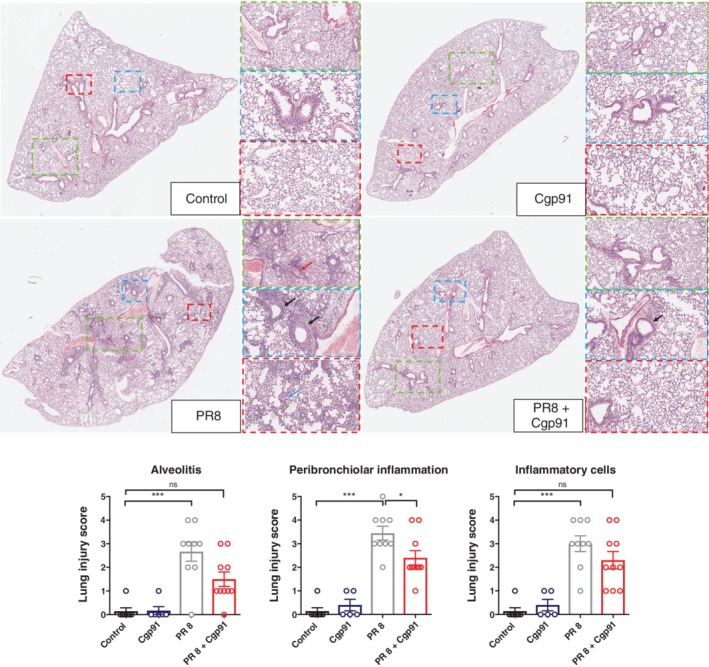
Lung histopathological stains reveal Cgp91ds‐TAT reduces airway inflammation in PR8‐infected mice. Histopathological analysis of lungs from WT C57Bl/6J mice treated daily via intranasal administration Cgp91ds‐TAT (0.2 mg/kg) or DMSO (2%; control) over a 4‐day period. Mice were infected with PR8 (500 PFU) or PBS control 1 day post initial drug treatment and analysed at Day 3 post‐infection. Representative H&E images displaying the inflammation in lung sections following H&E staining. Each sample was assigned a score of 0–5 for each individual mouse (higher numbers indicate increased disease severity), as assessed by two independent assessors. Sections were scored for alveolitis, inflammatory cell infiltrate and peribronchiolar inflammation. Magnifications of images are at ×1, ×3, ×6, ×10. The black arrows show peribronchial inflammation, red arrow perivascular inflammation and blue alveolitis. Data were expressed as mean ± SEM (control, *n* = 7; Cgp91ds‐TAT, *n* = 5; PR8, *n* = 9; PR8 + Cgp91ds‐TAT, *n* = 10). Statistical analysis was conducted using one‐way ANOVA followed by Tukey's post hoc test for multiple comparison tests. Statistical significance was taken where *P* < 0.05. *P < 0.05; ***P < 0.001. ANOVA, analysis of variance; Cgp91ds‐TAT, cholestanol‐conjugated gp91ds‐TAT; DMSO, dimethyl sulphoxide; HE, haematoxylin and eosin; PFU, plaque forming units; WT, wild type.

To assess the pathological changes associated with IAV infection, H&E staining and pathology scoring system was employed. The lung tissue of PR8‐infected mice displayed high amounts of peribronchiolar inflammation with extensive epithelial destruction compared to the lungs of uninfected control mice (Fig. 2). There was also evidence of intense intra‐alveolar inflammatory cellular infiltrates and significant perivascular inflammation when compared to the controls (Fig. 2). In comparison, Cgp91ds‐TAT‐treated mice displayed only relatively minor histological changes in response to PR8 infection. There was less alveolitis and epithelial denudation, and although there was still evidence of peribronchial inflammation, it did not appear to be as widespread (Fig. 2). There was also a reduction in perivascular inflammation (Fig. 2). Furthermore, Cgp91ds‐TAT alone did not cause any apparent adverse inflammation and the lung histology was similar to those of the uninfected controls (Fig. 2).

### Cgp91ds‐TAT reduces viral load and ROS generation

**Figure 3:**
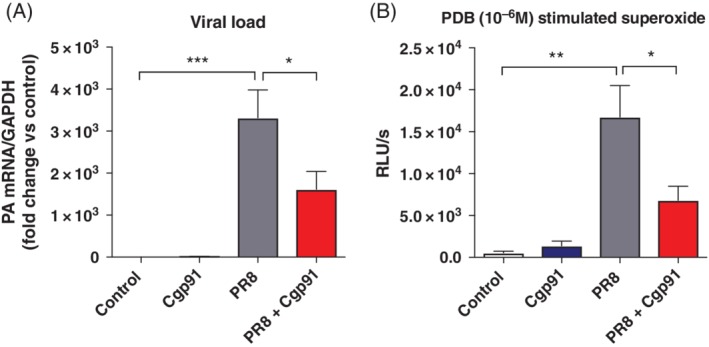
Cgp91ds‐TAT markedly reduces viral mRNA expression and ROS generation. WT C57Bl/6J mice (8–12 weeks) were treated daily via intranasal administration of Cgp91ds‐TAT (0.2 mg/kg) or DMSO (2%) control. Mice were intranasally infected with PR8 (500 PFU) or PBS control 1 day post initial drug treatment. (A) Quantitative PCR analysis in lung tissue of mRNA from the gene encoding polymerase of influenza virus strain PR8; results were presented relative to those of GAPDH mRNA. (B) BALF was collected for PDB (10^−6^ M)‐stimulated ROS production that was quantified by L‐O12 enhanced chemiluminescence. Data were expressed as mean ± SEM (control, *n* = 7; Cgp91ds‐TAT, *n* = 5; PR8, *n* = 9; PR8 + Cgp91ds‐TAT, *n* = 10). Statistical analysis was conducted using one‐way ANOVA test followed by Tukey's post hoc test for multiple comparison. Statistical significance was taken where *P* < 0.05. *P < 0.05; **P < 0.01; ***P < 0.001. ANOVA, analysis of variance; BALF, bronchoalveloar lavage fluid; Cgp91ds‐TAT, cholestanol‐conjugated gp91ds‐TAT; DMSO, dimethyl sulphoxide; PCR, polymerase chain reaction; PDB, phorbol dibutyrate; PFU, plaque forming units; RLU, relative light units; ROS, reactive oxygen species; WT, wild type.

Viral polymerase mRNA was detected in lung tissue in the PR8‐infected mice (Fig. 3A). Mice treated with Cgp91ds‐TAT displayed a significantly reduced amount of viral mRNA expression when compared to the virus control group (Fig. 3A). Importantly, the viral PA expression in naïve control and naïve Cgp91ds‐TAT‐treated mice was below the detection limit (Fig. 3A).

L‐012 enhanced chemiluminescence was used to measure ROS generation in cells from the BALF of mice. Cgp91ds‐TAT treatment of naïve‐uninfected inflammatory cells taken from mice did not alter the amount of ROS detected when compared to the uninfected control group. Cells from PR8‐infected mice exhibited a significant increase in the amount of ROS production when compared to the uninfected control group (Fig. 3B). BALF inflammatory cells taken from Cgp91ds‐TAT‐treated virus‐infected mice produced significantly lower amounts of ROS than the PR8‐infected control mice (Fig. 3B).

## DISCUSSION

IAV cause severe respiratory tract infections, which are associated with lung inflammation, excessive ROS production from NOX2 oxidase and significant lung pathology.^6^, ^7^, ^8^, ^12^, ^13^, ^14^, ^15^ The spatial restrictions of ROS strongly suggest that their site of production governs their site of action. Indeed, we have recently identified endosomes as key sites of ROS generation during influenza virus infections.^9^, ^16^ To this end, we custom‐modified the NOX2 oxidase peptide inhibitor gp91ds‐TAT to target these subcellular compartments and tested the therapeutic potential of localized NOX2 oxidase inhibition. Importantly, this novel endosome‐targeted inhibitor of NOX2 oxidase reduced the severity of various clinical outcomes associated with a low pathogenic IAV infection.^9^ This was characterized by a reduction in airway inflammation, lung injury and oxidative stress. In the present study, we utilized a high dose of the highly pathogenic IAV, that is PR8, and tested whether this endosome NOX2 oxidase targeting system had the capacity to reduce lung pathogenesis. Airway and lung parenchymal inflammation are important pathological characteristics of influenza virus infections, particularly those due to highly pathogenic strains, such as PR8. In the present study, PR8 infection caused significant changes to lung morphology characterized by extensive alveolitis, peribronchiolar inflammation and infiltrating inflammatory cells, together with airway epithelial denudation and perivascular inflammation. Strikingly, Cgp91ds‐TAT treatment attenuated all of these key parameters with marked reductions in alveolitis, suggesting that endosome ROS is a major contributor to the deleterious parenchymal inflammation observed during infections with highly pathogenic IAV.

Neutrophil influx is a hallmark feature of the host innate immune response to influenza virus infections^17^; however, there is conflicting evidence for what the ultimate role is for incoming neutrophils. For instance, neutrophil‐depleted mice exhibited exacerbated amounts of pulmonary inflammation and respiratory dysfunction during influenza virus infection,^18^ suggesting that these cells are critical for minimizing influenza virus‐induced pathology. By contrast, partial suppression of neutrophil infiltration was associated with a milder pathology and improved morbidity. Differences may also arise from different dose of virus used between studies, which may cause varied inflammatory responses. In this study, a high dose of PR8 virus resulted in a substantial increase in neutrophil influx into the airways of mice at Day 3 post‐infection. By contrast, Cgp91ds‐TAT treatment reduced airway inflammation, and it resulted in a partial ~50% reduction in neutrophil infiltration following PR8 infection. Although the role of neutrophils in influenza virus infections is debatable, our data suggest that a partial suppression of neutrophil infiltration combined with inhibition of ROS resulted in less overall lung pathology and also enhanced viral clearance. Importantly, Cgp91ds‐TAT had no effect on the infiltration of monocytes/macrophages and lymphocytes in the airways following PR8 infection. These preserved responses might be critical for containment and clearance of viral infection and for secondary bacterial superinfections and pneumonia.

ROS modulate a myriad of signalling networks in inflammatory cells such as macrophages to influence and shape the immune response to viruses such as influenza. We previously showed that endosomally generated ROS, most likely hydrogen peroxide (H_2_O_2_) by NOX2 oxidase, is a powerful negative regulator of Type I IFN production that was triggered by IAV as well as by activation of the TLR7 with imiquimod in isolated macrophages and in lung tissue in vivo.^9^ This suppressive effect of H_2_O_2_ on TLR7‐dependent IFN production was abrogated when the highly conserved and unique cysteine 98 (C98) of TLR7 was mutated to alanine, suggesting that the molecular target of H_2_O_2_ produced within the endosome is C98. Importantly and consistent with the spatial restrictions of ROS, C98 on TLR7 resides within the endosomal luminal side of the receptor and thus would be in the immediate vicinity of H_2_O_2_. Therefore, we suggest that inhibition of NOX2 oxidase by Cgp91ds‐TAT results in a larger Type I IFN production to IAV infection, leading to a stronger antiviral response against the virus to thereby reduce lung inflammation. Our findings are consistent with previous work that shows strong evidence for a reciprocal regulation of NOX2 and Type I IFN in humans and mice. In fact, an enhanced Type I IFN response signature that was accompanied by elevated autoantibody levels was identified in both mice and humans lacking functional NOX2 complex.^19^

Intriguingly, our study raises an important question: How does IAV escape the potential deleterious effects of ROS production in the endosomal compartment? Given that inhibition of endosome NOX2 oxidase ROS with Cg91ds‐TAT or with genetic deletion of NOX2 decreases viral load in the lung,^7^, ^9^ we hypothesize that ROS *per se* do not directly cause oxidative damage to the virus to prevent it from escaping the endosome. In contrast, it suggests that influenza virus actually utilize NOX2‐derived ROS to promote their replication and survival. Indeed, we and others have shown that viruses hijack the endocytic compartment to drive a TLR7‐dependent NOX2 oxidase‐dependent ROS process that suppresses Type I IFN responses, which is one of the most important antiviral mechanisms that enable the immune system to clear a viral infection.

Previous studies have touted the use of antioxidant therapies, such as N‐acetyl‐l‐cysteine (NAC) and vitamins including C and E for reducing influenza pathogenicity with some promising results.^20^, ^21^ Yet, this has not been proven to have a significant impact on either viral clearance or lung pathology. Given the rapid chemistry of ROS such as superoxide, H_2_O_2_ and peroxynitrite, all of which have been implicated in influenza pathology, we suggest that a better alternative is to specifically suppress ROS production at its source, rather than to use a generalized antioxidant strategy that relies on widespread distribution and downstream chemical inactivation.^9^, ^16^ Our data provide evidence that targeting endosomal ROS is an effective approach for preventing airway inflammation and lung injury to both mild and highly pathogenic IAV. The current findings demonstrate a clinical potential for endosome NOX2 inhibitors, particularly as a means for preventing influenza pathology. Whilst our study shows a clear potential for prevention of this inflammation to pandemic influenza by delivering an endosome NOX2 inhibitor prior to infection, it remains to be determined whether these inhibitors can limit the pathology when delivered post‐infection. This is certainly worthy of future investigation and is therefore a limitation of the current study. Important for these future studies is an examination of the effect of Cgp91ds‐TAT on the resolution of pandemic influenza which should include a comprehensive analysis of critical arms of both the adaptive and humoral immune systems. These studies will provide strong evidence for whether endosome NOX2 oxidase inhibitors have therapeutic potential in a post‐viral infection setting. In conclusion, the present study supports an emerging strategy that aims to suppress oxidative stress caused by IAV infection that could provide novel and effective approaches to avoid/alleviate influenza pathology.

**Abbreviations**
ANOVA: analysis of variance
BALF: bronchoalveloar lavage fluid
Cgp91ds‐TAT: cholestanol‐conjugated gp91dsTAT
DMSO: dimethyl sulphoxide
IAV: influenza A virus
IFN: interferon
NOX2: NADPH oxidase 2
PBS: phosphate‐buffered saline
PFU: plaque forming units
ROS: reactive oxygen species
TLR7: toll‐like receptor 7
WT: wild type

## Supporting information


**Visual Abstract** Pathogenic inflammation in response to Influenza A virus is promoted by endosomally located NOX2 oxidase.
