# Supplementary figures and images for: Novel endosomal NOX2 oxidase inhibitor ameliorates pandemic influenza A virus‐induced lung inflammation in mice
Source: Respirology. 2019 Mar 18;24(10):1011–7. doi: 10.1111/resp.13524 (PMC6972593; doi:10.1111/resp.13524)

## Slide 1
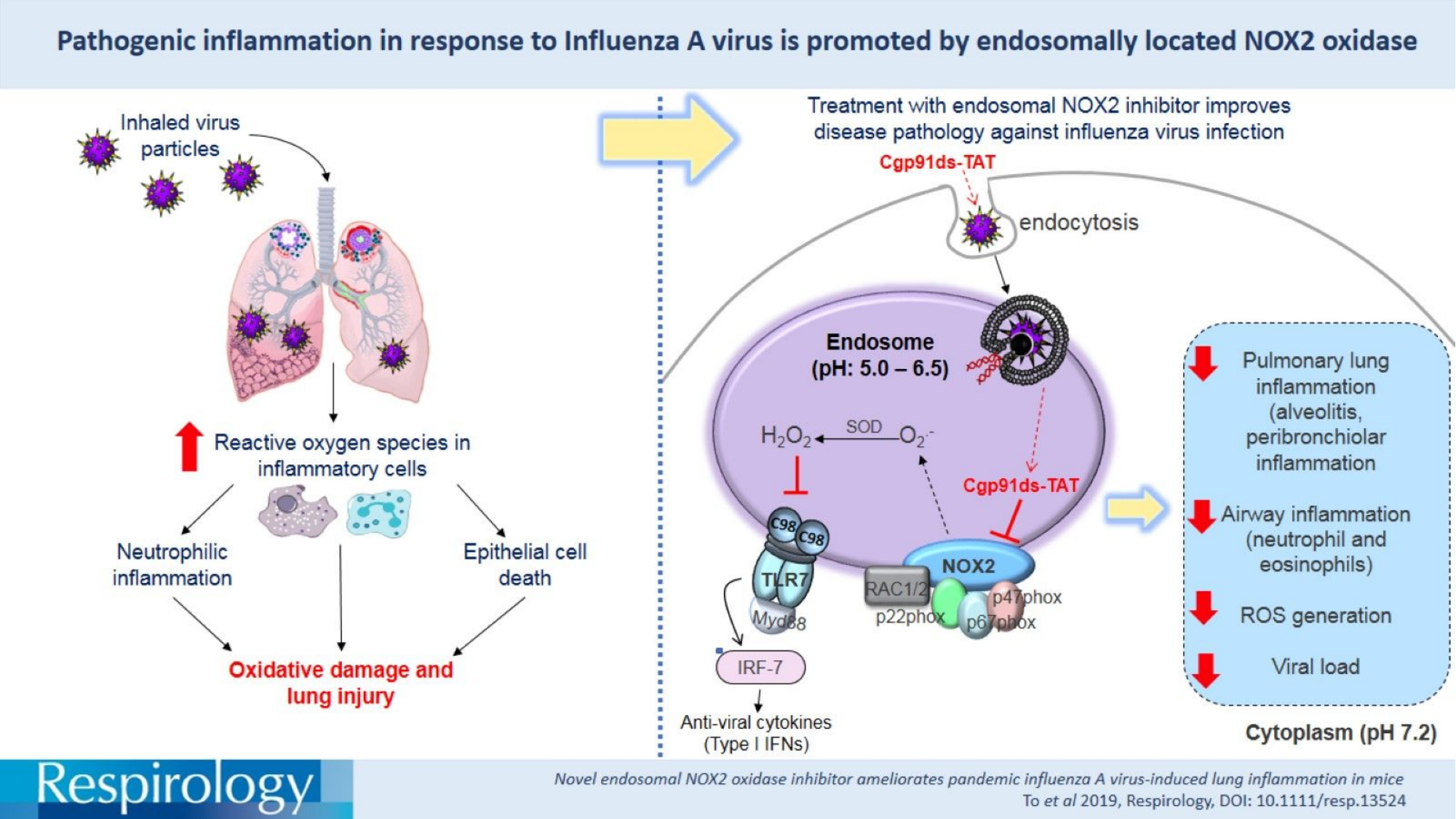

#

Supplement: Supplementary file 1 — Visual Abstract Pathogenic inflammation in response to Influenza A virus is promoted by endosomally located NOX2 oxidase. [file RESP-24-1011-s001.pptx]
